# Recurrent autoimmune hypophysitis treated with rituximab: a case report

**DOI:** 10.1186/s13256-021-03146-0

**Published:** 2021-12-15

**Authors:** Maria Kruse, Thomas Bastholm Olesen, Ljubo Markovic, Dorte Glintborg, Marianne Skovsager Andersen

**Affiliations:** 1 Department of Internal Medicine, Hospital of Littlebelt, Kolding, Denmark; 2 Department of Radiology, Odense University Hospital, Odense, Denmark; 3 Department of Endocrinology and Metabolism, Odense University Hospital, Odense, Denmark

**Keywords:** Autoimmune hypophysitis, Lymphocytic hypophysitis, Primary hypophysitis, CD20+ B-cells, Immunotherapy, Monoclonal antibody, Rituximab

## Abstract

**Background:**

Autoimmune hypophysitis is a rare condition that often results in enlargement of the pituitary gland and hypopituitarism due to inflammatory infiltration. Management of autoimmune hypophysitis can include long-term hormonal replacement and close control of the inflammatory pituitary mass. Mass-related symptoms in patients with autoimmune hypophysitis are treated with anti-inflammatory therapy, surgery, and/or radiotherapy.

**Case presentation:**

We present a 25-year-old White man with visual field defects of the right eye, headache, and weight loss. Magnetic resonance imaging showed a sellar mass, and the patient underwent transcranial surgery. Histopathology revealed autoimmune hypophysitis with predominantly CD20 positive B-cell infiltration. Progression of visual field defects necessitated postoperatively anti-inflammatory treatment with prednisolone. Azathioprine was initiated under gradual tapering of prednisolone with stable conditions at first, but relapse followed after dose reduction. Therefore, rituximab treatment was initiated, which resulted in regression of the pituitary mass. Rituximab treatment was discontinued after 25 months. The patient has continuously been in remission for 4 years after rituximab treatment was stopped.

**Conclusion:**

This case illustrates that rituximab might be an effective alternative treatment in B-cell predominant autoimmune hypophysitis.

## Background

Hypophysitis is a rare condition characterized by inflammatory infiltration of the pituitary gland. Hypophysitis can be divided into two main categories: primary hypophysitis (autoimmune, granulomatous, xanthomatous, immunoglobulin G4 (IgG4)-related, or necrotizing inflammation) and secondary hypophysitis. Primary hypophysitis has an incidence of ~ 1 in 9 million per year [1]. The most common form of primary hypophysitis is autoimmune hypophysitis (AH). AH has a strong female preponderance with the majority of cases identified during late pregnancy or postpartum. AH is confirmed histologically and immunohistochemically by lymphocytic infiltration (mostly T and B lymphocytes), plasma cells, histiocytes, and fibrosis [2, 3]. Secondary causes of hypophysitis include infectious and systemic diseases (such as systemic lupus erythematosus), sellar/parasellar lesions, and drug-therapy-induced hypophysitis [3–6].

Pituitary inflammation in hypophysitis usually results in enlargement of the pituitary gland with sellar compression and compression of the surrounding neurological structures including the optic chiasm. Therefore, patients often present with headache with or without nausea and visual disturbances [3]. The infiltrative inflammation of the pituitary gland can affect different parts of the pituitary gland, resulting in a broad symptomatology including anterior pituitary hormone deficiencies, hyperprolactinemia, and diabetes insipidus. AH is considered to have a predilection to adrenocorticotropic hormone (ACTH), thyroid-stimulating hormone (TSH), gonadotropin hormone, and growth hormone deficiencies. ACTH and TSH deficiencies are very frequent in the early stages of AH unlike pituitary adenomas, which results in increased risk of life-threatening adrenal insufficiency [4, 7].

Typically, in primary hypophysitis, magnetic resonance imaging (MRI) of the sella region shows an enlarged symmetric homogeneous pituitary gland, a thickened but not deviated stalk, and an intact sellar floor [7]. MRI findings cannot distinguish hypophysitis from the more common differential diagnoses of pituitary mass, that is, pituitary adenomas, germinomas, Langerhans cell histiocytosis, and metastases. Approximately 40% of cases of primary hypophysitis are misdiagnosed preoperatively [8]. Histopathology is the gold standard for diagnosing hypophysitis. However, biopsy of a pituitary mass is rarely performed due to the risks of the procedure [3, 9]. Unlike other autoimmune endocrine diseases, measurement of anti-pituitary antibodies is not suitable for diagnosing AH [4, 7, 8, 10–12]. Thus, a presumptive diagnosis of hypophysitis is often due to a combination of clinical findings, radiology, and biochemical results [3, 4, 6].

Treatment of AH consists of managing pituitary enlargement and substituting acute and persistent pituitary hormone deficiencies. Reduction of the pituitary enlargement is required in some cases where mass effects are present. In asymptomatic cases, conservative treatment with close follow-up is an alternative. Pituitary enlargement can be reduced by anti-inflammatory therapy, surgery, stereotactic radiotherapy, or a combination of these types of intervention. High-dose glucocorticoids are the basis of anti-inflammatory therapy. Steroid-sparing agents such as azathioprine (an anti-metabolic agent with immunosuppressive activity) are considered in the case of relapse with mass-related symptoms or increased mass size on MRI, need of long-term anti-inflammatory treatment, or intolerable adverse effects to glucocorticoids. Rituximab, a monoclonal antibody that selectively induces apoptosis in B-lymphocytes, is approved for various immune-mediated disorders and has been used to manage recurrent AH in a few previous cases [3, 9–11, 13–15].

## Case presentation

**Fig. 1:**
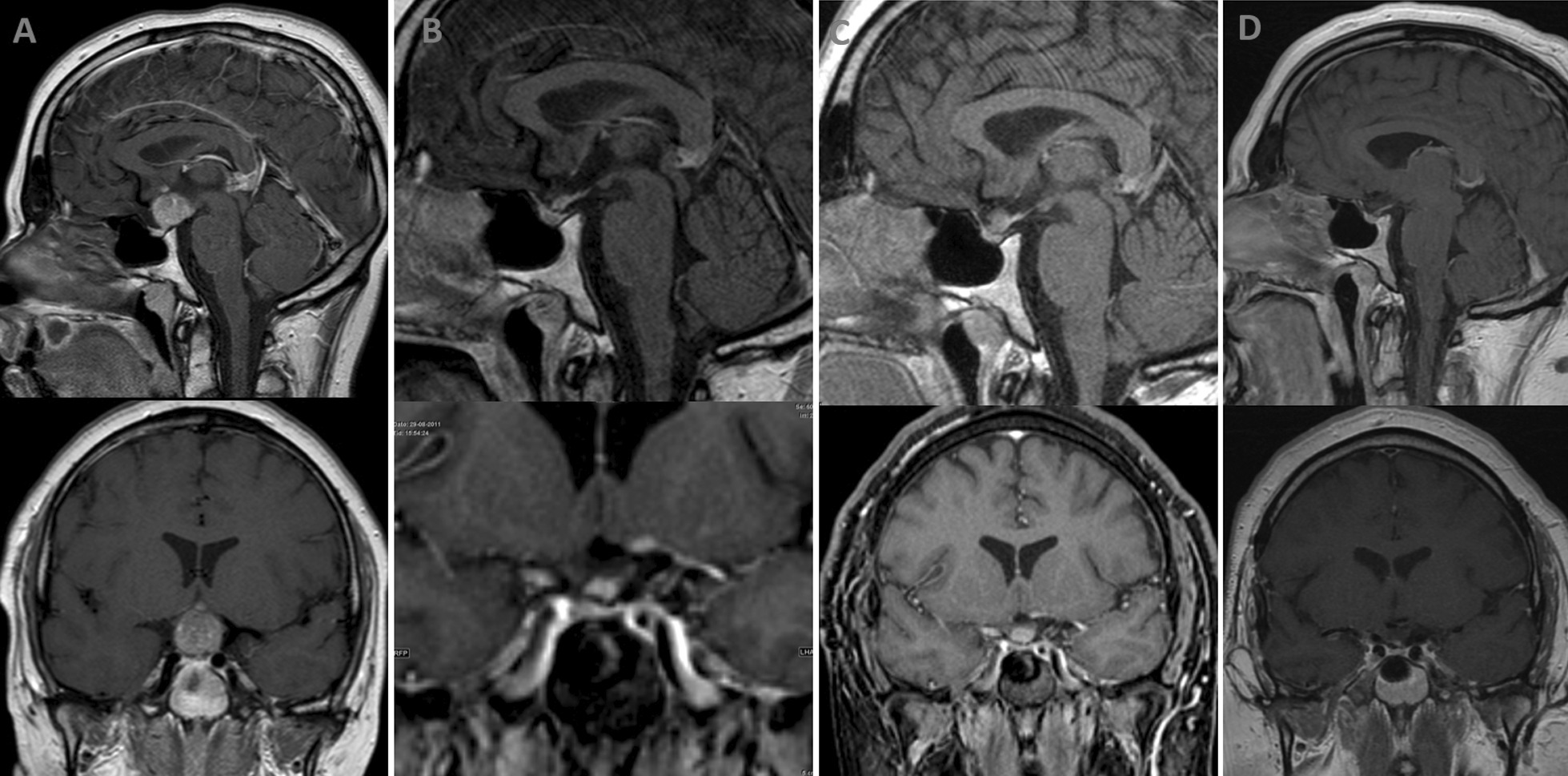
MRI scan showing pituitary enlargement in the T1-weighted sagittal (upper) and coronal section (lower). **A** Initial MRI before surgery. **B** Post-surgery with remnant pituitary tissue located just below the optic chiasm. **C** Relapse under treatment with azathioprine and prednisolone. **D** Almost complete regression of pituitary mass after rituximab treatment

A previously healthy 25-year-old White man presented with a 1-year history of blurred vision in the right eye, headache, and weight loss of about 30 kg. On physical examination, he had a body mass index of 28 kg/m^2^ and visual field defects in the right eye. Initial MRI showed a lightly spotted heterogeneous 19 × 16 × 19 mm tumor located suprasellarly, close to the pituitary gland with no ingrowth or association with the pituitary gland (Fig. 1A). Endocrine evaluation revealed secondary hypogonadism with low follicle-stimulating hormone, luteinizing hormone, and testosterone but intact thyrotroph and lactotroph axes. An ACTH test was performed with a subnormal cortisol response (30-minute cortisol 248 nmol/L, reference > 420 nmol/L) and low plasma ACTH concentration, indicating secondary adrenal insufficiency, and the patient started treatment with hydrocortisone. The somatotroph axis was not evaluated. Visual field measured by perimetry revealed visual field defects in the right eye.

The tumor was initially diagnosed based on MRI as a craniopharyngioma for which reason the patient was referred to craniotomy as standard treatment. At surgery, the tumor appeared gray and reddish, and was capsulated and fragile with easy bleeding, unlike craniopharyngiomas with sharp, irregular borders, which have a tendency to adhere to vital neurovascular structures and often consist of cystic and/or solid parts [16]. The pituitary tumor was partially resected, and a small amount of capsule remnant underneath the optic chiasm could not be surgically removed. Perioperative frozen section histological examination described the tumor as a possible malignant lymphoma due to lymphocytic infiltration.

The patient was transferred to the Department of Endocrinology at Odense University Hospital for further diagnostic workup and management of pituitary hormone deficiencies. Postoperatively, the patient was still affected by headache and visual deficits on ophthalmologic assessment. MRI showed regression of the pituitary mass (9 × 6 × 8 mm), but remnant pituitary tissue was located just below the optic chiasm (Fig. 1B). Pituitary hormone insufficiency of all anterior axes and diabetes insipidus was diagnosed, and treatment with hydrocortisone, levothyroxine, testosterone, growth hormone, and desmopressin was implemented.

Endocrine evaluation did not support a hormone-producing adenoma. Sellar lymphoma is a very rare disease often related to immunodeficiency, for example HIV-related, or seen in immunocompetent patients who are over 60 years old [17]. Metastasis was not suspected; the patient did not suffer from cancer in another location. Workup for infectious and rheumatologic etiologies was not indicative of secondary hypophysitis, nor was drug-related infiltration of the pituitary gland a possible differential diagnosis. Final histological diagnosis showed lymphoid reaction with predominant CD20 positive B-cell infiltration and no sign of significant IgG4 involvement. The patient was diagnosed with autoimmune hypophysitis.

Ten months postoperatively, the patient reported impaired vision. MRI showed that the remnant tissue lifted and exerted pressure at the optic chiasm. Perimetry confirmed progression of visual field defects in the right eye and now involvement of the left eye. Because of progression of visual field defects, treatment with glucocorticoid pulse therapy was initiated: methylprednisolone 500 mg infusion for 3 days, followed by reduced prednisolone doses, that is, 37.5 mg per day. Follow-up MRI after 1 month of glucocorticoid treatment (11 months postoperatively) demonstrated significant reduction of the pituitary mass and decreased pressure at the optic chiasm. Perimetry was unchanged. Treatment with prednisolone continued for 3 months.

The patient had intolerable adverse effects to glucocorticoid treatment with excessive weight gain of about 50 kg and cushingoid features. The need for long-term treatment led to initiation of steroid-sparing treatment with 150 mg azathioprine per day under gradual tapering of prednisolone (13 months postoperatively). Visual field defects improved, and repeated MRI showed additional regression of the remnant pituitary mass. Perimetry revealed stable conditions. Due to stable condition over 26.5 months, treatment with azathioprine was reduced. Control MRI after one and a half month showed recurrence with increasing mass in sella turcica causing mass effect on the optic chiasm. Treatment was resumed: azathioprine 100 mg per day in combination with prednisolone 37.5 mg per day.

Despite additional 11 months treatment with azathioprine and prednisolone treatment (52 months postoperatively), control MRI showed further progression with increasing pituitary mass size (11 × 6 × 7 mm) reaching the optic chiasm (Fig. 1C). Treatment with rituximab was initiated: 1000 mg infusions 14 days apart, repeated every 12 months. This resulted in almost complete regression of pituitary mass (3 × 2 × 7 mm) evaluated by MRI (Fig. 1D). Rituximab treatment was discontinued after 25 months (77 months postoperatively) because of stable disease. To date, the patient has been followed up for ten and a half years postoperatively. According to control MRI 4 years after end of treatment with rituximab, he is in sustained remission with unchanged pituitary mass size and no affection of the optic chiasm.

## Discussions and conclusion

AH progresses through initial stages of inflammation, lymphocyte infiltration, edema, and enlargement to fibrosis and subsequent atrophy [11]. Treatment with glucocorticoid pulse therapy has shown good response. In this case, the initial response to glucocorticoid treatment was good; however, the patient had intolerable adverse effects that led to change in treatment. Gutenberg *et al*. showed reduction in pituitary size in 75% of patients with AH during pre- or postsurgical glucocorticoid treatment [18]. However, relapse of AH is common and treatment with glucocorticoids, especially long-term treatment, is associated with a high risk of side effects (weight gain, psychiatric symptoms, edema, avascular bone necrosis, and diabetes mellitus). Honegger *et al*. showed a recurrence rate at 38% and found a high rate of significant adverse effects under glucocorticoid pulse therapy (63% of involved patients) [15].

If steroid treatment is discontinued either due to mass-related symptoms or due to intolerable adverse effects, other immunosuppressive therapy is considered. In this case, azathioprine was initiated under gradual tapering of prednisolone with stable conditions at first, but relapse followed after dose reduction. Azathioprine has been the most commonly used immunosuppressive agent; however, immunosuppressive or biological treatment directed at the predominant cell type involved may increase treatment efficacy [13, 14, 19]. The infiltration of the pituitary gland in AH is typically a mixture of T and B cells, often with one cell type being more predominant than the other. Most lympholytic drugs primarily target T cells, such as azathioprine, whereas rituximab specifically binds to the CD20 antigen on B lymphocytes, inducing apoptosis [13, 14]. Additionally, rituximab may reduce the antigen presentation, leading to T-cell inactivation [20, 21]. The potential effect of histologically guided treatment of AH is illustrated in a case reported by Xu *et al*. [14] The patient was initially treated with prednisolone and methotrexate without effect and afterwards treated with infliximab (a monoclonal antibody that antagonizes tumor necrosis factor alpha). Due to recurrence of pituitary enlargement, a transsphenoidal biopsy was performed, showing CD20 positive B lymphocytes as the dominant cell type. Subsequently, treatment with rituximab resulted in long-term remission [14].

Steroid-sparing treatment seems promising as a useful addition to glucocorticoids. There is no indication for rituximab in AH yet; however, it should be considered as an alternative treatment. Table 1 provides a brief summary of the papers reporting use of steroid-sparing therapies in AH, adding to the previous summary by Joshi *et al*. [3]. The case reports indicate good response to azathioprine and rituximab in patients with progression or recurrence as well as adverse effects to glucocorticoids.

**Table 1:** Brief summary of cases using azathioprine or rituximab in autoimmune hypophysitis

Refs.	Case characteristics	Treatment	Histology	Outcome
Xu *et al*. [14]	54-year-old female with uveitis, scleritis, and diabetes insipidus	Infliximab and rituximab	CD20-positive B lymphocytes as the dominant cell type	Remission (follow-up 18 months)
Schreckinger *et al*. [13]	41-year-old female with visual loss in the left eye and sellar mass	Relapse at high-dose methylprednisolone. Changed to rituximab	CD20-positive B lymphocytes as the dominant cell type	Stable clinical and radiological improvement
De Bellis *et al*. [20]	36-year-old male with primary immune thrombocytopenia (ITP) and hypogonadotropic hypogonadism	Rituximab	Clinical diagnosis of autoimmune hypophysitis No histopathology	Complete remission of ITP and autoimmune hypophysitis
Lecube *et al*. [19]	53-year-old male with frontal headache, diplopia, and diabetes insipidus	Relapse at prednisolone. Changed to azathioprine	Mixed B and T lymphocytes	Complete resolution
Curto *et al*. [22]	38-year-old male with diplopia, blurred vision, and headaches	Relapse at high-dose methylprednisolone. Changed to azathioprine	Typical features of autoimmune hypophysitis	Decrease in pituitary mass
Lu *et al*. [23]	22-year-old female with headache, diplopia, left eye ptosis, and lactation	Relapse after transsphenoidal surgery. High-dose methylprednisolone and azathioprine	Lymphocytic, histiocytic, and neutrophil infiltration consistent with AH	Uneventful pregnancy and delivery Long-term remission
Wang *et al*. [24]	70-year-old female with diabetes insipidus and adenopituitary function deficiency	Relapse after glucocorticoid treatment High-dose methylprednisolone and azathioprine	Clinical diagnosis of autoimmune hypothalamitis No histopathology	Remission on treatment
Yang *et al*. [25]	1) 22-year-old female with headache and diplopia 2) 70-year-old female with diabetes insipidus and dry mouth 3) 32-year-old female with diabetes insipidus, menstrual disorder, headache, and dizziness	Recurrence after surgery and prednisolone respectively. Changed to high-dose methylprednisolone followed by prednisolone and azathioprine for all cases	Lymphocytic infiltration in pituitary tissue in case 1 No other histopathology	Complete resolution on MRI with endocrine recovery in case 1 and 3 and permanent hypopituitarism in case 2
Katsivali *et al*. [26]	48-year-old female with headache, muscle weakness, and occlusion of both internal carotid arteries	Relapse when prednisolone tapering Due to adverse effects, treatment was changed to azathioprine	Clinically suspected AH No histopathology	Complete resolution and restored pituitary function
Curto *et al*. [27]	38-year-old male with diplopia, ophthalmoplegia, and headache	Initial response on high-dose methylprednisolone. Changed to azathioprine	Lymphocytes with CD20-positive B cells and CD3-positive T cells	No response to treatment

The current case illustrates that, in treatment of AH where surgery and traditional immunosuppressive therapy is insufficient or intolerable, monoclonal antibody-directed therapy such as rituximab can be a preferable alternative.

## Data Availability

Not applicable.

## Abbreviations

AH: Autoimmune hypophysitis
ACTH: Adrenocorticotropic hormone
TSH: Thyroid-stimulating hormone
MRI: Magnetic resonance imaging
